# Cultural capital and health literacy among urban communities in Malaysia: A cross-sectional study

**DOI:** 10.1371/journal.pone.0352662

**Published:** 2026-06-29

**Authors:** Nurul Izyan Husna Ibrahim, Abdul Hafiz Ab Rahman, Azlina Abdullah, Shamarina Shohaimi

**Affiliations:** 1 Centre for Research in Development, Social and Environment, Faculty of Social Sciences and Humanities, Universiti Kebangsaan Malaysia, Bangi, Selangor, Malaysia; 2 Department of Biology, Faculty of Science, Universiti Putra Malaysia, Serdang, Selangor, Malaysia; Universitas Prima Indonesia, INDONESIA

## Abstract

Health literacy is an important determinant of health outcomes, yet many approaches continue to emphasize individual cognitive skills while giving limited attention to the sociocultural resources that shape how people engage with health information. This study examined the association between cultural capital and multiple dimensions of health literacy among urban communities in Malaysia. A cross-sectional study was conducted among 325 adults residing in selected urban communities in Ipoh, Perak. A cluster-based approach was used to identify study locations, followed by community-based convenience recruitment within selected clusters. Cultural capital was assessed across embodied, objectified and institutionalized dimensions, while health literacy was measured using the validated HLS-M-Q18 instrument, which captures access, understanding, evaluation and application of health information. All constructs demonstrated good internal consistency (Cronbach alpha = 0.822 to 0.931). Spearman’s rank correlation was used for bivariate analysis due to non-normality, while multiple linear regression estimated the independent contribution of each form of cultural capital to each health literacy domain. Cultural capital was significantly associated with all dimensions of health literacy (p < 0.001). Institutionalized cultural capital was the strongest predictor of access (β = 0.298, p < 0.001) and understanding (β = 0.305, p < 0.001), whereas embodied cultural capital was the only significant predictor of evaluation (β = 0.537, p < 0.001) and the strongest predictor of application (β = 0.359, p < 0.001). Cultural capital explained between 28.2% and 41.9% of the variance across outcomes. Differences were also observed across education and income levels. These findings indicate that health literacy is not determined by access to information alone but is shaped by broader social and cultural resources. Public health interventions should therefore move beyond information provision and strengthen the experiential and contextual capacities that enable individuals to evaluate and apply health information in everyday life.

## Introduction

Health literacy is commonly defined as the ability to access, understand, evaluate and apply health information in ways that support informed decision-making [1]. Higher levels of health literacy have been associated with improved health behaviours, greater use of preventive services and better disease management [2]. Early conceptualizations emphasized functional skills, particularly reading and comprehension, as central to navigating health information [3]. While these skills remain important, they are insufficient for explaining how individuals engage with increasingly complex, dynamic and often contradictory information environments.

Recent work has reframed health literacy as a socially situated capability shaped by broader structural and cultural conditions [4]. Individuals engage with health information within specific social contexts, where interpretation and use are influenced by accumulated resources, experiences and dispositions. As a result, individuals with similar levels of access or formal education may differ substantially in their ability to assess credibility, interpret meaning and translate information into action [4]. This distinction is important because contemporary health communication increasingly requires individuals not only to obtain information, but also to judge whether information is reliable, relevant and applicable to their circumstances.

Cultural capital provides a useful framework for understanding these differences. As conceptualized by Bourdieu, cultural capital exists in embodied, objectified and institutionalized forms, each shaping how individuals acquire, interpret and use knowledge [5]. Within the context of health literacy, cultural capital has been used to explain how social and cultural resources influence health-related knowledge, practices and inequalities [6,7]. Institutionalized cultural capital refers to formal educational qualifications, while objectified cultural capital refers to cultural goods and material resources and embodied cultural capital refers to durable dispositions, knowledge and ways of acting [5**]**. In this study, these forms are interpreted in relation to health literacy, for example formal education may support the understanding of written health information, material and informational resources may increase exposure to health information and embodied dispositions may shape confidence, communication practices and the use of experiential knowledge in health-related decision-making [1,3].

This framework allows health literacy to be understood as more than an individual cognitive skill. Formal education may support reading and comprehension, while material resources may increase exposure to health information. However, the ability to evaluate whether information is credible and to apply it in everyday life may depend more strongly on embodied forms of knowledge, including confidence, practical judgement and accumulated experience. Although cultural capital has been associated with health inequalities, its role across specific domains of health literacy remains underexplored [6].

Access to health information has expanded through health systems, traditional media, social media and mobile health platforms [8]. However, access to information alone does not ensure that individuals can understand, appraise and apply health information in ways that improve health behaviours or outcomes [2,8]. Individuals exposed to similar information may differ in how they interpret, evaluate and act on it, indicating that effective engagement with health information cannot be explained by access alone [2].

This distinction is particularly relevant in contemporary information environments characterized by high levels of exposure and variability in information quality. Individuals are required not only to access information but also to critically evaluate its credibility and relevance, especially in the presence of misinformation. In such settings, health literacy depends on the interaction between information access, social position, cultural resources and everyday experience.

Malaysia provides an important setting for examining this issue because access to health information has expanded substantially in recent years, particularly in urban settings, alongside high levels of internet and mobile device use reported nationally [9]. Despite this, disparities in health literacy persist, suggesting that access alone does not ensure effective engagement [1,10]. Urban populations are exposed to diverse sources of information, including digital media, interpersonal networks and formal healthcare systems, yet variation in how information is interpreted and used remains evident [4].

The specific research gap addressed in this study is the limited empirical evidence on how different forms of cultural capital relate to different domains of health literacy in urban Malaysian communities. Existing studies have largely emphasized education, digital access, or individual literacy skills, with less attention to the sociocultural resources that may shape higher-order competencies such as evaluation and application. This study therefore examines the association between cultural capital and multiple dimensions of health literacy among urban communities in Malaysia. Specifically, it assesses how embodied, objectified and institutionalized forms of cultural capital relate to access, understanding, evaluation and application of health information.

## Materials and methods

### Study design and setting

A cross-sectional study was conducted among adults residing in selected urban communities in Ipoh, Perak, Malaysia, to examine the association between cultural capital and health literacy. The selected communities were urban residential areas with mixed socioeconomic characteristics, including variation in educational attainment, employment status, income and exposure to health information sources. This setting was suitable for the study because cultural capital is closely related to social position and may vary across educational and socioeconomic groups.

Ipoh was selected because it represents an urban Malaysian setting with a sizeable and socially diverse population [11]. Urban residents may encounter health information through multiple formal and informal sources, including healthcare professionals, the internet, family members, television and community-based activities [12]. This makes Ipoh an appropriate setting for examining health literacy, particularly how individuals access, understand, appraise and apply health information in relation to healthcare, disease prevention and health promotion [13,14]. Given the variation in education, income, employment and material resources within urban populations, the setting also provided a suitable context for examining cultural capital as a sociocultural determinant of health literacy [11,13**]**.

### Study population and sampling

The study population comprised adults aged 18 years and above residing in the selected urban communities. Urban localities were defined as clusters based on geographical and administrative boundaries and eligible individuals within these clusters were approached for participation. Because no comprehensive household-level or individual-level sampling frame was available, participants were recruited using community-based convenience sampling through direct engagement with individuals who were present at the time of data collection. Eligibility criteria included being aged 18 years and above, residing in the selected areas and being able to understand and respond to the questionnaire. Individuals who were unable to complete the questionnaire due to cognitive or language limitations were excluded. A total of 325 respondents were included in the final analysis. As recruitment was based on convenience sampling within selected clusters, the sample should not be interpreted as statistically representative of all urban residents in Perak.

The sample size was initially guided by the conventional single-proportion formula for cross-sectional studies, using a 95% confidence level, an expected proportion of 50% and a 5% margin of error, which gives a minimum target of 384 respondents [15]. A total of 325 respondents were included in the final analysis after data screening. Although this was below the initial survey target, the sample remained adequate for the planned multiple regression analyses. Each regression model included three main predictors: embodied, objectified and institutionalized cultural capital. Based on Green’s recommendation of N > 50 + 8m, where m represents the number of predictors, the minimum required sample for three predictors was 74 respondents [16]. The final sample therefore exceeded the minimum requirement for the planned regression models, although the findings should be interpreted in light of the non-probability sampling approach.

### Participant recruitment

Participants were recruited through face-to-face engagement within residential neighbourhoods. Trained research team members approached potential participants, explained the study objectives, and invited eligible individuals to participate. To reduce selection bias, recruitment was conducted across multiple locations within each cluster and at different times of the day, including weekdays and weekends. Participation was voluntary and no incentives were provided. Written informed consent was obtained prior to participation.

### Data collection procedures

Data were collected between February 2025 and August 2025 using a structured, self-administered questionnaire. Where necessary, clarification was provided by the research team to ensure accurate understanding of the items. The questionnaire was administered in a language familiar to participants to minimize misinterpretation. All responses were recorded anonymously. Participants were informed of the study purpose, their rights and their ability to withdraw at any time without consequence.

### Measures

#### Cultural capital.

Cultural capital was operationalized based on the conceptual framework proposed by Bourdieu [5**]**, encompassing embodied, objectified and institutionalized capital. Items measuring cultural capital were developed by mapping each dimension of cultural capital to observable indicators relevant to health information engagement. Embodied cultural capital reflected internalized dispositions, confidence, communication practices and experiential knowledge related to health information. Objectified cultural capital captured access to material and informational resources, including digital devices and health-related information sources. Institutionalized cultural capital was represented by formal educational attainment.

The item development process involved adapting Bourdieu’s conceptual categories to the health information context. For example, embodied cultural capital was represented by indicators related to confidence in discussing health information, experience in interpreting health advice and familiarity with using health information in everyday decision-making. Objectified cultural capital was represented by access to health-related materials and digital or informational resources. Institutionalized cultural capital was represented by formal educational qualifications. The items were reviewed by the research team for conceptual relevance and contextual suitability before data collection. The questionnaire was pre-tested among a small group of participants to assess clarity, wording and acceptability. Composite scores for each dimension were calculated as the mean of the corresponding items, with higher scores indicating higher levels of cultural capital.

#### Health literacy.

Health literacy was assessed using the HLS-M-Q18 instrument, a validated Malaysian adaptation of the European Health Literacy Survey questionnaire [17**]**, based on the conceptual framework proposed by Sørensen et al. [1**]**. The instrument measures four domains of health literacy: access, understanding, evaluation and application of health information. Domain-specific scores were calculated as mean values, with higher scores indicating higher levels of health literacy.

#### Reliability assessment.

Internal consistency of all constructs was assessed using Cronbach’s alpha. All variables demonstrated good to excellent reliability (α = 0.822–0.931) [18**]** and no items were removed. Although internal consistency was satisfactory, reliability was not interpreted as sufficient evidence of full construct validity for the cultural capital measures. This issue is acknowledged in the limitations.

### Statistical analysis

All analyses were conducted using IBM SPSS Statistics Version 30. Data were checked for completeness prior to analysis and no substantial missing data were observed. Descriptive statistics were used to summarize respondent characteristics and study variables. Normality was assessed using the Shapiro-Wilk test [19]. As the study variables were not normally distributed (p < 0.001), Spearman’s rank correlation was used to examine bivariate associations between cultural capital and health literacy domains.

Multiple linear regression analyses were conducted to assess the independent association between cultural capital and health literacy. Embodied, objectified and institutionalized cultural capital were entered as independent variables, while the four health literacy domains, namely access, understanding, evaluation and application, were analysed as dependent variables in separate regression models. Linear regression was retained for multivariable analysis because the objective was to estimate the independent contribution of each form of cultural capital while reporting standardized coefficients and explained variance. Regression assumptions were assessed prior to interpretation, including linearity, residual distribution, homoscedasticity, multicollinearity and influential observations. Multicollinearity was assessed using variance inflation factor and tolerance values [20]. Unstandardized coefficients (B), standardized beta coefficients (β), 95% confidence intervals and coefficients of determination (R²) were reported. Differences across sociodemographic characteristics were examined using Mann-Whitney U and Kruskal-Wallis tests [21]. Statistical significance was set at p < 0.05.

### Ethical considerations

Ethical approval was obtained from the Ethics Committee for Research Involving Human Subjects, Universiti Putra Malaysia (JKEUPM) (Reference No: JKEUPM-2024–1099). All participants provided written informed consent prior to data collection. Participation was voluntary and respondents were informed of their right to withdraw at any time without consequence. All data were collected anonymously and treated confidentially. The study did not involve minors.

## Results

### Sample characteristics

A total of 325 respondents were included in the analysis. Females comprised 59.4% (n = 193) of the sample, while males accounted for 40.6% (n = 132). The majority of respondents were aged between 18 and 35 years (56.0%, n = 182), followed by those aged 36–50 years (31.1%, n = 101) and 51 years and above (12.9%, n = 42).

In terms of ethnicity, 61.8% (n = 201) were Malay, 21.2% (n = 69) Indian, 13.8% (n = 45) Chinese, and 3.1% (n = 10) from other ethnic groups. Educational attainment varied across secondary education (SPM) (16.4%, n = 53), pre-university qualifications (STPM/STAM/Diploma) (35.1%, n = 114), bachelor’s degree (35.1%, n = 114), and postgraduate qualifications (8.9%, n = 29).

With respect to employment, 36.0% (n = 117) were employed in the government sector, 23.7% (n = 77) in the private sector, 19.4% (n = 63) were students and 20.9% (n = 68) were in other categories. Monthly income varied, with 37.3% (n = 121) earning RM1001 to RM3000, 23.1% (n = 75) earning RM3001 to RM5000, 12.3% (n = 40) earning RM5001 and above, 3.1% (n = 10) earning RM1000 and below, and 24.3% (n = 79) reporting no income. These distributions indicate that the sample included adults from mixed educational, occupational and income backgrounds. The demographic characteristics of the respondents are summarized in Table 1.

**Table 1:** Demographic characteristics of respondents (N = 325).

Variable	Category	n (%)
Gender	Male	132 (40.6)
	Female	193 (59.4)
Age	18–35	182 (56.0)
	36–50	101 (31.1)
	51 and above	42 (12.9)
Ethnicity	Malay	201 (61.8)
	Chinese	45 (13.8)
	Indian	69 (21.2)
	Others	10 (3.1)
Education	Secondary (SPM)	53 (16.4)
	Pre-university (STPM/STAM/Diploma)	114 (35.1)
	Bachelor’s degree	114 (35.1)
	Postgraduate	29 (8.9)
Employment	Government	117 (36.0)
	Private	77 (23.7)
	Student	63 (19.4)
	Others	68 (20.9)
Income (RM)	Not working	79 (24.3)
	1000 and below	10 (3.1)
	1001–3000	121 (37.3)
	3001–5000	75 (23.1)
	5001 and above	40 (12.3)

SPM = Sijil Pelajaran Malaysia; STPM = Sijil Tinggi Persekolahan Malaysia; STAM = Sijil Tinggi Agama Malaysia.

### Reliability of measures

All constructs demonstrated good to excellent internal consistency. Cronbach’s alpha values ranged from 0.822 to 0.931, indicating satisfactory reliability across all variables. The reliability results for all study variables are presented in Table 2.

**Table 2:** Reliability of study variables.

Variable	Cronbach’s alpha
Embodied cultural capital	0.829
Objectified cultural capital	0.840
Institutionalized cultural capital	0.852
Access	0.931
Understanding	0.822
Evaluation	0.868
Application	0.885

### Correlation analysis

Spearman correlation analysis showed significant positive associations between all dimensions of cultural capital and all domains of health literacy (all p < 0.01). These findings indicate that higher levels of cultural capital were consistently associated with higher health literacy scores across access, understanding, evaluation and application. The correlation coefficients are presented in Table 3.

**Table 3:** Spearman correlation between cultural capital and health literacy.

Variable	Access	Understanding	Evaluation	Application
Embodied	0.425**	0.481**	0.598**	0.605**
Objectified	0.470**	0.498**	0.466**	0.581**
Institutionalized	0.503**	0.573**	0.439**	0.566**

** p < 0.01.

Embodied cultural capital was strongly correlated with evaluation (ρ = 0.598) and application (ρ = 0.605) and moderately correlated with access (ρ = 0.425) and understanding (ρ = 0.481). Institutionalized cultural capital showed the strongest correlation with understanding (ρ = 0.573), followed by application (ρ = 0.566) and access (ρ = 0.503). Objectified cultural capital demonstrated moderate correlations across all domains.

### Regression analysis

Multiple linear regression analyses were conducted to examine whether the three forms of cultural capital were independently associated with the four domains of health literacy. Embodied, objectified and institutionalized cultural capital were entered as independent variables, while access, understanding, evaluation and application were analysed as separate dependent variables. Unstandardized coefficients (B) indicate the expected change in each health literacy domain score for a one-unit increase in the predictor, while standardized coefficients (β) were used to compare the relative strength of predictors within each model. All four regression models were statistically significant (p < 0.001), with explained variance ranging from 28.2% for access to 41.9% for application. The regression results are summarized in Table 4.

**Table 4:** Multiple regression analysis predicting health literacy.

Outcome	Predictor	B (95% CI)	β	p-value
Access	Embodied	0.129 (0.030, 0.228)	0.167	0.011
	Objectified	0.103 (0.001, 0.206)	0.144	0.047
	Institutionalized	0.198 (0.114, 0.283)	0.298	<0.001
Understanding	Embodied	0.169 (0.075, 0.264)	0.214	<0.001
	Objectified	0.134 (0.037, 0.232)	0.183	0.007
	Institutionalized	0.207 (0.126, 0.288)	0.305	<0.001
Evaluation	Embodied	0.510 (0.398, 0.622)	0.537	<0.001
	Objectified	0.038 (−0.078, 0.153)	0.043	0.521
	Institutionalized	0.080 (−0.016, 0.175)	0.098	0.101
Application	Embodied	0.273 (0.185, 0.361)	0.359	<0.001
	Objectified	0.133 (0.043, 0.224)	0.188	0.004
	Institutionalized	0.127 (0.052, 0.201)	0.194	<0.001

A differentiated pattern was observed across the health literacy domains. For access, institutionalized cultural capital was the strongest predictor (B = 0.198, 95% CI [0.114, 0.283], β = 0.298, p < 0.001), followed by embodied cultural capital (B = 0.129, 95% CI [0.030, 0.228], β = 0.167, p = 0.011) and objectified cultural capital (B = 0.103, 95% CI [0.001, 0.206], β = 0.144, p = 0.047). A similar pattern was found for understanding, where institutionalized cultural capital again showed the strongest association (B = 0.207, 95% CI [0.126, 0.288], β = 0.305, p < 0.001), followed by embodied cultural capital (B = 0.169, 95% CI [0.075, 0.264], β = 0.214, p < 0.001) and objectified cultural capital (B = 0.134, 95% CI [0.037, 0.232], β = 0.183, p = 0.007). These models explained 28.2% and 37.2% of the variance in access and understanding, respectively.

In contrast, evaluation was associated only with embodied cultural capital (B = 0.510, 95% CI [0.398, 0.622], β = 0.537, p < 0.001), while objectified cultural capital (B = 0.038, 95% CI [−0.078, 0.153], β = 0.043, p = 0.521) and institutionalized cultural capital (B = 0.080, 95% CI [−0.016, 0.175], β = 0.098, p = 0.101) were not significant predictors. The evaluation model explained 39.4% of the variance. For application, embodied cultural capital was also the strongest predictor (B = 0.273, 95% CI [0.185, 0.361], β = 0.359, p < 0.001), followed by institutionalized cultural capital (B = 0.127, 95% CI [0.052, 0.201], β = 0.194, p < 0.001) and objectified cultural capital (B = 0.133, 95% CI [0.043, 0.224], β = 0.188, p = 0.004). This model explained the largest proportion of variance (R² = 0.419).

Overall, institutionalized cultural capital was most strongly associated with the more functional domains of health literacy, namely access and understanding. In contrast, embodied cultural capital was more strongly associated with the higher-order domains of evaluation and application. This suggests that while formal qualifications may support the ability to obtain and comprehend health information, the capacity to judge credibility and use information in everyday life may depend more strongly on internalized knowledge, confidence and practical experience.

### Differences across sociodemographic characteristics

Non-parametric tests were conducted to examine differences in health literacy across sociodemographic characteristics. No significant differences were observed between males and females across all domains of health literacy (p > 0.05). Significant differences were observed across age groups for evaluation and application (p < 0.05), but not for access or understanding. Educational attainment was significantly associated with all domains of health literacy (p < 0.001). Income level was also significantly associated with all domains (p < 0.01). The results of these sociodemographic comparisons are summarized in Table 5.

**Table 5:** Differences in health literacy across sociodemographic characteristics.

Variable	Access	Understanding	Evaluation	Application
Gender	ns	ns	ns	ns
Age	ns	ns	*	*
Education	***	***	***	***
Income	***	**	***	***

Differences were assessed using Mann-Whitney U and Kruskal-Wallis tests. ns = not significant; *p < 0.05; **p < 0.01; ***p < 0.001

These findings support the interpretation that health literacy differences in this sample were more closely patterned by educational and socioeconomic position than by gender. The presence of differences across education and income groups also reinforces the relevance of examining cultural and material resources in relation to health literacy.

## Discussion

This study examined how embodied, objectified and institutionalized cultural capital were associated with four domains of health literacy among urban communities in Malaysia. The findings show that health literacy is not a uniform capability shaped by a single resource. Instead, different domains of health literacy were associated with different forms of cultural capital. Institutionalized cultural capital was most strongly associated with access and understanding, whereas embodied cultural capital was most strongly associated with evaluation and application. This pattern suggests that health literacy should be understood as a differentiated and socially situated process, rather than as a simple progression from information access to behaviour change.

The association between institutionalized cultural capital and access and understanding is consistent with established evidence linking education to functional health literacy and health outcomes [2,3]. Formal education may improve familiarity with written materials, institutional communication and technical language. In practical terms, individuals with higher educational attainment may be better equipped to locate health information, read official materials, understand medical instructions and navigate formal health systems. However, the findings also show that education alone did not explain the ability to evaluate health information. This is important because contemporary health decisions increasingly require individuals to judge conflicting claims, identify credible sources and decide whether general advice is relevant to their own circumstances.

The stronger association between embodied cultural capital and evaluation and application provides a key theoretical contribution. Embodied cultural capital reflects accumulated experience, communication practices, confidence and practical judgement [5]. These resources may be especially important when individuals encounter ambiguous or conflicting health information. Evaluation requires more than reading ability. It requires people to assess credibility, compare sources, draw on prior experience and decide whether information can be trusted. Application requires another step: translating information into action within the constraints of family responsibilities, income, work, social norms and access to services. The findings therefore suggest that higher-order health literacy is not merely cognitive but is also experiential and socially embedded.

This interpretation aligns with the broader conceptualization of health literacy as a social determinant of health [4**]** but extends it by showing that specific domains of health literacy may have different sociocultural foundations. Access and understanding were more closely linked to institutionalized cultural capital, while evaluation and application were more closely linked to embodied cultural capital. This distinction helps explain why improving access to health information does not automatically lead to better decision-making or behaviour change. People may have access to information and may understand its literal content, yet still lack the confidence, social support, or practical experience needed to judge its relevance and apply it in everyday life.

The findings are particularly relevant in digitally saturated information environments. Digital platforms have increased exposure to health information, but they have also increased exposure to misinformation, commercialized health claims and contradictory advice. Previous digital health literacy research has shown that access to online information does not guarantee the ability to use it effectively [22,23]. The present study adds that this gap is not only technological or cognitive, but also sociocultural. Access to digital tools and information sources may increase exposure, but critical appraisal depends on resources that are accumulated through experience, social interaction and repeated engagement with health systems and health information.

The lack of significant associations between institutionalized and objectified cultural capital and evaluation further reinforces this interpretation. Material resources and formal education may help individuals obtain information, but they may not be sufficient for judging credibility. This is consistent with evidence that individuals often struggle to evaluate online health information even when they have access to digital resources [24]. In this study, embodied cultural capital was the only significant predictor of evaluation, indicating that experiential knowledge and practical judgement may be central to critical health literacy.

The association between income and all domains of health literacy further situates the findings within broader structural conditions. Income may shape access to devices, internet connectivity, transport, healthcare services, time and the ability to act on health advice. Even when individuals understand health information, material constraints may limit whether they can apply it. This supports the view that health literacy is embedded within broader social and economic conditions, rather than being solely an individual attribute [6].

The absence of gender differences suggests that health literacy disparities in this sample were not primarily patterned by gender. Instead, differences were more evident across education and income groups. Age-related differences in evaluation and application may reflect accumulated experience over time, although this interpretation should be treated cautiously because the cross-sectional design does not allow developmental conclusions. These sociodemographic patterns reinforce the importance of locating health literacy within social and cultural contexts.

The broader public health implication is that interventions focusing only on access or information provision are unlikely to reduce disparities in health literacy. In contemporary public health, individuals are not only required to find health information. They must also decide which information is credible, which advice is relevant and how recommendations can be applied within their own social and material circumstances. Health literacy interventions should therefore place greater emphasis on experiential learning, trusted community communication, practical decision-making and context-specific support. Community-based approaches that allow people to discuss, test and apply health information in relation to their lived realities may be more effective than interventions that simply distribute information.

This study contributes to health literacy research by showing that the domains of health literacy are not shaped equally by the same resources. By applying Bourdieu’s concept of cultural capital to the four domains of health literacy, the study provides a more differentiated explanation of why access to information does not necessarily translate into critical appraisal or practical application. This contribution is especially relevant for urban populations in Malaysia, where access to information is expanding but social and cultural differences continue to shape how information is interpreted and used.

### Limitations

Several limitations should be acknowledged. First, the cross-sectional design prevents causal inference and the findings should be interpreted as associations rather than causal effects. Second, although study locations were identified through a cluster-based approach, participants within clusters were recruited using community-based convenience sampling. This may have introduced selection bias because participation depended on availability and willingness to participate at the time of data collection. Third, the findings may not be generalizable to all urban communities in Perak or Malaysia. Fourth, cultural capital was measured using context-specific indicators adapted for this study. Although the measures demonstrated satisfactory internal consistency, the study did not conduct a full psychometric validation of the cultural capital indicators. Future studies should examine dimensionality and construct validity using exploratory and confirmatory factor analysis and should validate the instrument across different Malaysian populations. Fifth, the final sample of 325 respondents fell short of the minimum target of 384 calculated using Cochran’s single‑proportion formula; while this was adequate for the planned regression analyses, the shortfall may reduce the precision of our estimates and limit statistical power. Finally, self-reported data may be subject to response bias, particularly for items involving confidence, information practices and perceived ability to use health information.

## Conclusion

This study shows that health literacy is not determined solely by access to information or formal education. Instead, it is shaped by a combination of institutionalized, objectified, embodied and economic resources. Formal education was more strongly associated with access and understanding, while embodied cultural capital was more strongly associated with evaluation and application. These findings indicate that different domains of health literacy depend on different forms of cultural and social resources.

The findings highlight a critical limitation in current health literacy approaches that emphasize access and functional skills. Improving access to information alone is unlikely to reduce disparities in health literacy if individuals do not also have the experiential, social and contextual resources needed to judge and apply information. Public health strategies should therefore move beyond information provision and strengthen the social and experiential conditions that enable people to interpret, evaluate and use health information in everyday life.

## Supporting information

S1 Data Dataset. (SAV)
