# Supplementary material for: Cultural capital and health literacy among urban communities in Malaysia: A cross-sectional study
Source: PLoS One. 2026 Jun 29;21(6):e0352662. doi: 10.1371/journal.pone.0352662 (PMC13313365; doi:10.1371/journal.pone.0352662)
Supplement: Attachment — Submitted filename: Response to Reviewers.docx [file pone.0352662.s003.docx]

Response to Reviewers

**Manuscript title:**

**Cultural capital and health literacy among urban communities in Malaysia: A cross-sectional study**

**Dear Academic Editor and Reviewers,**

We sincerely thank the reviewers for their insightful comments and constructive feedback. We have carefully revised the manuscript in response to these comments. Below we provide a detailed, point‑by‑point response. Changes have been incorporated throughout the manuscript as indicated.

**General overview of revisions**

We revised the introduction to provide a clearer explanation of Bourdieu’s forms of cultural capital and how each relates to health literacy. We sharpened the theoretical framework and research rationale, clarified the sampling strategy and sample size justification, described the study setting in greater detail and added a discussion of measurement development and limitations. The statistical analysis section was expanded to explain the choice of tests and assessment of regression assumptions. We also enhanced the discussion by linking findings to existing literature and policy implications. The references have been cross‑checked and updated.

# Responses to editorial assessment questions

## 1. Technical soundness and support for conclusions

**Reviewer comment:** Both reviewers indicated that the manuscript was partly technically sound and that the data partly supported the conclusions.

**Response:** We have revised the manuscript to ensure that the conclusions are more directly supported by the data. We now describe the study as cross-sectional and associative throughout the manuscript and avoid causal language. The conclusion has been revised to state that cultural capital was associated with health literacy domains, rather than implying causation. The limitations section now clearly acknowledges the cross-sectional design, convenience recruitment within clusters, limited generalisability, measurement limitations and the final sample size shortfall relative to the initial survey target.

## 2. Statistical analysis

**Reviewer comment:** Both reviewers indicated that the statistical analysis was not performed appropriately and rigorously.

**Response:** We have expanded the statistical analysis section to explain the analytical choices and assumption checks. Normality was assessed using the Shapiro-Wilk test and Spearman’s rank correlation was used for bivariate analyses because the study variables were not normally distributed. Multiple linear regression was retained for the multivariable analyses because the aim was to estimate the independent contribution of embodied, objectified and institutionalised cultural capital to each health literacy domain while reporting standardised coefficients and explained variance. We also added that regression assumptions were assessed, including linearity, residual distribution, homoscedasticity, multicollinearity and influential observations. Unstandardised coefficients, standardised beta coefficients, 95% confidence intervals, p-values and R² values are now reported.

## 3. Data availability

**Reviewer comment:** Both reviewers indicated that the data underlying the manuscript findings had not been made fully available.

**Response:** We have revised the Data Availability Statement. The anonymised dataset underlying the findings will be provided as Supporting Information. The dataset contains no personally identifiable information and includes the variables required to reproduce the descriptive, reliability, correlation and regression analyses reported in the manuscript.

## 4. Presentation and language

**Reviewer comment:** Reviewer 1 indicated that the manuscript was not sufficiently clear, while Reviewer 2 considered the manuscript clearly written but noted that it lacked a stronger high-impact narrative.

**Response:** We have edited the manuscript for clarity, flow and standard academic English. We clarified key concepts, reduced repetitive phrasing and improved transitions between the introduction, methods, results and discussion. We also strengthened the narrative by emphasising the broader public health significance of examining cultural capital as a sociocultural determinant of health literacy, particularly in contemporary information environments where access to information alone may not ensure meaningful evaluation or application.

# Reviewer 1

## Comment 1: Explanation of cultural capital

**Reviewer comment:** The manuscript introduces the concept of cultural capital based on Bourdieu’s framework; however, the explanation remains highly abstract and insufficiently contextualized for a multidisciplinary readership. Although embodied, objectified and institutionalized cultural capital are briefly defined, the manuscript does not adequately explain how these constructs practically relate to health literacy in everyday settings. Readers outside sociology or health promotion may find it difficult to comprehend the meaning and application of these concepts within the study context. More concrete examples and clearer operational explanations are needed to improve accessibility and interpretability of the findings.

**Response:** We agree with this comment. The introduction has been revised to explain the three forms of cultural capital in more concrete terms. Institutionalised cultural capital is now explained as formal educational qualifications; objectified cultural capital as material and informational resources, including digital devices and health information sources; and embodied cultural capital as durable dispositions, confidence, communication practices, experiential knowledge and practical judgement. We also added examples of how these forms may relate to health literacy, such as formal education supporting comprehension of written health information, material resources increasing exposure to health information and embodied dispositions shaping confidence and judgement in health-related decision-making.

## Comment 2: Theoretical framework

**Reviewer comment:** The study lacks a robust theoretical framework underpinning the proposed relationship between cultural capital and health literacy. While Bourdieu’s theory is cited, the manuscript does not sufficiently explain the theoretical mechanisms linking each form of cultural capital to the domains of health literacy. The rationale for why institutionalized cultural capital should influence access and understanding, or why embodied cultural capital predicts evaluation and application, is underdeveloped. As a result, the study appears largely descriptive and correlational rather than theory-driven.

**Response:** We have strengthened the theoretical framing in the introduction and discussion. The revised manuscript now explains why institutionalised cultural capital may be more closely linked to access and understanding, while embodied cultural capital may be more important for evaluation and application. We explain that access and understanding may depend more on formal education and familiarity with written or institutional information, whereas evaluation and application require practical judgement, confidence, prior experience and the ability to decide whether information is credible and relevant to one’s circumstances. This makes the manuscript more theory-driven and less purely descriptive.

## Comment 3: Study rationale and Malaysian context

**Reviewer comment:** The rationale for conducting the study requires further strengthening. Although the introduction discusses increasing access to health information and persistent disparities in health literacy, the manuscript does not clearly establish the specific research gap that justifies this study within the Malaysian context. The reasons for focusing specifically on urban communities in Perak are not adequately explained. The manuscript would benefit from a clearer justification regarding the public health importance, contextual relevance, and potential implications of examining cultural capital within urban Malaysian populations.

**Response:** We have revised the introduction to clarify the research gap. The manuscript now explains that although access to health information has expanded in Malaysia, disparities in health literacy persist. Existing studies have largely focused on education, digital access or individual literacy skills, with less attention to the sociocultural resources that may shape higher-order health literacy competencies such as evaluation and application. We also strengthened the justification for the study setting. Ipoh, Perak is now described as an urban Malaysian setting with a socially diverse population and variation in education, income, employment and material resources. This provides a relevant context for examining how cultural capital relates to health literacy.

## Comment 4: Sampling rigour and representativeness

**Reviewer comment:** The methodological approach raises concerns regarding sampling rigor and representativeness. The manuscript describes the use of a “multistage cluster sampling approach”; however, participant recruitment within clusters was ultimately conducted using convenience sampling. This creates ambiguity regarding the actual sampling strategy and may overstate the methodological robustness of the study. The use of convenience sampling limits representativeness and introduces potential selection bias, which should be more explicitly acknowledged and discussed.

**Response:** We agree that this required clarification. The methods section has been revised to state that urban localities were defined as clusters based on geographical and administrative boundaries, but that eligible participants within these clusters were recruited through community-based convenience sampling because no comprehensive household-level or individual-level sampling frame was available. We also added a clear statement that the sample should not be interpreted as statistically representative of all urban residents in Perak. The limitations section now acknowledges that this recruitment approach may have introduced selection bias because participation depended on availability and willingness to participate at the time of data collection.

## Comment 5: Sample size justification

**Reviewer comment:** The manuscript does not provide any evidence or rationale explaining how the sample size of 325 respondents was determined. No power analysis, sample size formula, effect size estimation, or reference to regression sample size requirements is presented. Given that multiple regression analyses were conducted, an appropriate justification for the adequacy of the sample size is necessary to support the validity of the statistical findings.

**Response:** We have added a sample size justification in the methods section. The initial target sample size was guided by Cochran’s single-proportion formula for cross-sectional studies, using a 95% confidence level, an expected proportion of 50% and a 5% margin of error, which gave a minimum target of 384 respondents. The final sample included 325 respondents after data screening. We acknowledge that this was below the initial target. However, the sample remained adequate for the planned multiple regression analyses, as each model included three predictors and Green’s recommendation of N > 50 + 8m requires a minimum of 74 respondents for three predictors. We have also added this issue to the limitations section by stating that the final sample of 325 respondents was smaller than the minimum target of 384 calculated with Cochran’s formula; while adequate for the planned regression analyses, the shortfall may reduce the precision of estimates and limit statistical power.

## Comment 6: Study setting

**Reviewer comment:** The description of the study setting lacks sufficient detail. The term “selected urban communities” is repeatedly used without adequately describing the characteristics of these communities. It remains unclear whether the sample included urban poor populations, middle-income groups, highly educated residents, or mixed socioeconomic backgrounds. Since cultural capital is closely linked to social and economic positioning, clearer contextual description of the study population is essential for interpretation and transferability of the findings.

**Response:** We have expanded the “Study design and setting” section. The selected communities are now described as urban residential areas with mixed socioeconomic characteristics, including variation in educational attainment, employment status, income and exposure to health information sources. We also explain that this diversity is important because cultural capital is closely linked to social position and may vary across educational and socioeconomic groups. The demographic table and narrative results further describe the sample in terms of age, gender, ethnicity, education, employment and income.

## Comment 7: Operationalisation and validation of cultural capital

**Reviewer comment:** The operationalization of cultural capital requires further clarification and validation. Although the authors state that measurement items were developed based on the conceptual framework and adapted to the study context, there is insufficient information regarding item development procedures, construct validity assessment, dimensionality testing, or validation processes beyond Cronbach’s alpha values. Reliability alone is insufficient to establish the validity of a newly operationalized construct, particularly for a complex sociological concept such as cultural capital.

**Response:** We have expanded the measures section to describe how cultural capital was operationalised. The revised manuscript explains that items were developed by mapping Bourdieu’s conceptual categories to observable indicators relevant to health information engagement. Embodied cultural capital included indicators related to confidence in discussing health information, experience interpreting health advice and familiarity with using health information in everyday decision-making. Objectified cultural capital represented access to health-related materials and digital or informational resources, while institutionalised cultural capital represented formal educational qualifications. The items were reviewed by the research team for conceptual relevance and contextual suitability and were pre-tested for clarity, wording and acceptability. We also clarified that Cronbach’s alpha demonstrates internal consistency but does not establish full construct validity. The limitations section now states that future studies should examine dimensionality and construct validity using exploratory and confirmatory factor analysis and validate the instrument across different Malaysian populations.

## Comment 8: Presentation of results

**Reviewer comment:** The presentation of the results is statistically organized but conceptually difficult to follow. The manuscript does not consistently distinguish between independent and dependent variables in a manner that is easily understandable for general readers. While regression tables are presented, the narrative interpretation remains repetitive and fragmented. Greater clarity in explaining the analytical model and the relationships between variables would improve readability and comprehension.

**Response:** We have revised the results section to improve conceptual clarity. The manuscript now explicitly states that embodied, objectified and institutionalised cultural capital were entered as independent variables, while access, understanding, evaluation and application were analysed as dependent variables in separate regression models. We also explain that unstandardised coefficients represent the expected change in the outcome for a one-unit increase in the predictor, while standardised coefficients allow comparison of relative predictor strength within each model. The narrative now focuses on the differentiated pattern across health literacy domains rather than merely listing statistical values.

## Comment 9: Discussion and interpretation

**Reviewer comment:** The discussion section tends to restate statistical findings rather than critically interpreting them within broader theoretical and public health contexts. Although the authors attempt to connect the findings to sociocultural dimensions of health literacy, the interpretation remains somewhat superficial. Stronger integration with existing literature, clearer theoretical implications and deeper critical discussion are needed to enhance the scholarly contribution of the manuscript.

**Response:** We have substantially revised the discussion. The revised discussion interprets the findings in relation to Bourdieu’s concept of cultural capital and broader health literacy theory. We explain that institutionalised cultural capital may support access and understanding because formal education improves familiarity with written health information and institutional communication. We also explain that embodied cultural capital may be more important for evaluation and application because these domains require confidence, practical judgement, prior experience and the ability to assess credibility and relevance. We further discuss the implications for digitally saturated information environments, where access to information does not necessarily translate into critical appraisal or behaviour change. The public health implications have also been expanded to emphasise the need for interventions that move beyond information provision and strengthen experiential learning, trusted communication and context-specific support.

# Reviewer 2

## Comment 1: Writing quality and contribution

**Reviewer comment:** This manuscript is professional and clearly articulated. It follows the standard conventions of academic English quite well. The narrative structure is logical, successfully guiding the reader through the theoretical application of Bourdieu’s cultural capital within the specific context of Malaysian urban health literacy. While the prose is technically sound and free of major grammatical hurdles, it reads more like a standard research report than a high-impact discovery. It lacks the sharp, synthesis-driven storytelling that usually characterizes papers in top-tier journals. Instead, the text leans on descriptive phrasing that catalogs the findings rather than pushing a compelling or provocative new argument.

**Response:** We appreciate this constructive assessment. We have revised the manuscript to strengthen the broader narrative and make the contribution clearer. The introduction now positions the study within the problem of persistent health literacy disparities despite expanding access to health information. The discussion now emphasises that health literacy is not shaped by access alone but by sociocultural resources that affect people’s ability to interpret, evaluate and apply information. We believe this revised framing presents a clearer contribution to public health by showing why interventions that focus only on information access may be insufficient.

## Comment 2: Incremental nature of the study

**Reviewer comment:** The clarity of the writing is certainly a strength because it makes the research easy to follow, but it does not manage to mask the study's incremental nature. The authors have done a solid job of presenting their data, but the writing does not offer the kind of big picture perspective or breakthrough narrative required to move the needle in global public health discourse. Ultimately, while the paper is linguistically precise and meets the basic requirements for an academic publication, it lacks the stylistic spark and transformative depth expected for a high-impact submission.

**Response:** We have revised the manuscript to clarify the specific contribution of the study. Rather than presenting cultural capital as a general predictor of health literacy, we now emphasise the differentiated pattern across domains. The finding that institutionalised capital was more strongly associated with access and understanding, while embodied capital was more strongly associated with evaluation and application, provides a more nuanced account of why access to information does not necessarily lead to effective use of health information. We have also highlighted the relevance of this finding for digital and urban health contexts, where people are exposed to abundant health information but still require confidence, experience and practical judgement to evaluate and apply it.

We would like to thank the Academic Editor and reviewers again for their valuable comments. We believe that the revisions have strengthened the theoretical foundation, methodological transparency, statistical reporting and overall contribution of the manuscript. We hope that the revised manuscript will be deemed suitable for publication in PLOS One.

Sincerely,

Abdul Hafiz Ab Rahman

Corresponding Author

On behalf of all authors
