# Supplementary material for: Cultural capital and health literacy among urban communities in Malaysia: A cross-sectional study
Source: PLoS One. 2026 Jun 29;21(6):e0352662. doi: 10.1371/journal.pone.0352662 (PMC13313365; doi:10.1371/journal.pone.0352662)
Supplement: Peer review history

# Peer review: Decision Letter 0

**DOI:** 10.1371/journal.pone.0352662.r001

**Authors:** Kristiawan Indriyanto

12 May 2026

PONE-D-26-16700

Cultural capital and health literacy among urban communities in Malaysia: A cross-sectional study

PLOS One

Dear Dr. Ab Rahman,

Thank you for submitting your manuscript to PLOS ONE. After careful consideration, we feel that it has merit but does not fully meet PLOS ONE’s publication criteria as it currently stands. Therefore, we invite you to submit a revised version of the manuscript that addresses the points raised during the review process.

==============================

Please follow the reviewers' comments to improve the quality of your manuscript.

==============================

Please submit your revised manuscript by Jun 26 2026 11:59PM. If you will need more time than this to complete your revisions, please reply to this message or contact the journal office at plosone@plos.org. When you're ready to submit your revision, log on to https://www.editorialmanager.com/pone/ and select the 'Submissions Needing Revision' folder to locate your manuscript file.

Please include the following items when submitting your revised manuscript:

- A letter that responds to each point raised by the academic editor and reviewer(s). You should upload this letter as a separate file labeled 'Response to Reviewers'.
- A marked-up copy of your manuscript that highlights changes made to the original version. You should upload this as a separate file labeled 'Revised Manuscript with Track Changes'.
- An unmarked version of your revised paper without tracked changes. You should upload this as a separate file labeled 'Manuscript'.

If you would like to make changes to your financial disclosure, please include your updated statement in your cover letter. Guidelines for resubmitting your figure files are available below the reviewer comments at the end of this letter.

If applicable, we recommend that you deposit your laboratory protocols in protocols.io to enhance the reproducibility of your results. Protocols.io assigns your protocol its own identifier (DOI) so that it can be cited independently in the future. For instructions see: https://journals.plos.org/plosone/s/submission-guidelines#loc-laboratory-protocols. Additionally, PLOS ONE offers an option for publishing peer-reviewed Lab Protocol articles, which describe protocols hosted on protocols.io. Read more information on sharing protocols at https://plos.org/protocols?utm_medium=editorial-email&utm_source=authorletters&utm_campaign=protocols.

As the corresponding author, your ORCID iD is verified in the submission system and will appear in the published article. PLOS supports the use of ORCID, and we encourage all coauthors to register for an ORCID iD and use it as well. Please encourage your coauthors to verify their ORCID iD within the submission system before final acceptance, as unverified ORCID iDs will not appear in the published article. *Only*  the individual author can complete the verification step; PLOS staff *cannot*  verify ORCID iDs on behalf of authors.

We look forward to receiving your revised manuscript.

Kind regards,

Kristiawan Indriyanto

Academic Editor

PLOS One

Journal requirements:

When submitting your revision, we need you to address these additional requirements.

1. Please ensure that your manuscript meets PLOS ONE's style requirements, including those for file naming. The PLOS ONE style templates can be found at

https://journals.plos.org/plosone/s/file?id=wjVg/PLOSOne_formatting_sample_main_body.pdf and

https://journals.plos.org/plosone/s/file?id=ba62/PLOSOne_formatting_sample_title_authors_affiliations.pdf

2. In the online submission form, you indicated that [Insert text from online submission form here].

All PLOS journals now require all data underlying the findings described in their manuscript to be freely available to other researchers, either 1. In a public repository, 2. Within the manuscript itself, or 3. Uploaded as supplementary information.

This policy applies to all data except where public deposition would breach compliance with the protocol approved by your research ethics board. If your data cannot be made publicly available for ethical or legal reasons (e.g., public availability would compromise patient privacy), please explain your reasons on resubmission and your exemption request will be escalated for approval.

If the reviewer comments include a recommendation to cite specific previously published works, please review and evaluate these publications to determine whether they are relevant and should be cited. There is no requirement to cite these works unless the editor has indicated otherwise.

[Note: HTML markup is below. Please do not edit.]

Reviewers' comments:

Reviewer's Responses to Questions

**Comments to the Author**

1. Is the manuscript technically sound, and do the data support the conclusions?

The manuscript must describe a technically sound piece of scientific research with data that supports the conclusions. Experiments must have been conducted rigorously, with appropriate controls, replication, and sample sizes. The conclusions must be drawn appropriately based on the data presented.

Reviewer #1: Partly

Reviewer #2: Partly

**********

2. Has the statistical analysis been performed appropriately and rigorously?

Reviewer #1: No

Reviewer #2: No

**********

3. Have the authors made all data underlying the findings in their manuscript fully available?

The PLOS Data policy requires authors to make all data underlying the findings described in their manuscript fully available without restriction, with rare exception (please refer to the Data Availability Statement in the manuscript PDF file). The data should be provided as part of the manuscript or its supporting information, or deposited to a public repository. For example, in addition to summary statistics, the data points behind means, medians and variance measures should be available. If there are restrictions on publicly sharing data—e.g. participant privacy or use of data from a third party—those must be specified.

Reviewer #1: No

Reviewer #2: No

**********

4. Is the manuscript presented in an intelligible fashion and written in standard English?

PLOS ONE does not copyedit accepted manuscripts, so the language in submitted articles must be clear, correct, and unambiguous. Any typographical or grammatical errors should be corrected at revision, so please note any specific errors here.

Reviewer #1: No

Reviewer #2: Yes

**********

5. Review Comments to the Author

Please use the space provided to explain your answers to the questions above. You may also include additional comments for the author, including concerns about dual publication, research ethics, or publication ethics. (Please upload your review as an attachment if it exceeds 20,000 characters)

Reviewer #1: This is an interesting study that provides significant findings regarding health literacy and cultural capital. However, there are several issues that need to be improved to strengthen the overall quality of the work.

• The manuscript introduces the concept of cultural capital based on Bourdieu’s framework; however, the explanation remains highly abstract and insufficiently contextualized for a multidisciplinary readership. Although embodied, objectified, and institutionalized cultural capital are briefly defined, the manuscript does not adequately explain how these constructs practically relate to health literacy in everyday settings. Readers outside sociology or health promotion may find it difficult to comprehend the meaning and application of these concepts within the study context. More concrete examples and clearer operational explanations are needed to improve accessibility and interpretability of the findings.

• The study lacks a robust theoretical framework underpinning the proposed relationship between cultural capital and health literacy. While Bourdieu’s theory is cited, the manuscript does not sufficiently explain the theoretical mechanisms linking each form of cultural capital to the domains of health literacy. The rationale for why institutionalized cultural capital should influence access and understanding, or why embodied cultural capital predicts evaluation and application, is underdeveloped. As a result, the study appears largely descriptive and correlational rather than theory-driven

• The rationale for conducting the study requires further strengthening. Although the introduction discusses increasing access to health information and persistent disparities in health literacy, the manuscript does not clearly establish the specific research gap that justifies this study within the Malaysian context. The reasons for focusing specifically on urban communities in Perak are not adequately explained. The manuscript would benefit from a clearer justification regarding the public health importance, contextual relevance, and potential implications of examining cultural capital within urban Malaysian populations.

• The methodological approach raises concerns regarding sampling rigor and representativeness. The manuscript describes the use of a “multistage cluster sampling approach”; however, participant recruitment within clusters was ultimately conducted using convenience sampling. This creates ambiguity regarding the actual sampling strategy and may overstate the methodological robustness of the study. The use of convenience sampling limits representativeness and introduces potential selection bias, which should be more explicitly acknowledged and discussed.

• The manuscript does not provide any evidence or rationale explaining how the sample size of 325 respondents was determined. No power analysis, sample size formula, effect size estimation, or reference to regression sample size requirements is presented. Given that multiple regression analyses were conducted, an appropriate justification for the adequacy of the sample size is necessary to support the validity of the statistical findings

• The description of the study setting lacks sufficient detail. The term “selected urban communities” is repeatedly used without adequately describing the characteristics of these communities. It remains unclear whether the sample included urban poor populations, middle-income groups, highly educated residents, or mixed socioeconomic backgrounds. Since cultural capital is closely linked to social and economic positioning, clearer contextual description of the study population is essential for interpretation and transferability of the findings.

• The operationalization of cultural capital requires further clarification and validation. Although the authors state that measurement items were developed based on the conceptual framework and adapted to the study context, there is insufficient information regarding item development procedures, construct validity assessment, dimensionality testing, or validation processes beyond Cronbach’s alpha values. Reliability alone is insufficient to establish the validity of a newly operationalized construct, particularly for a complex sociological concept such as cultural capital.

• The presentation of the results is statistically organized but conceptually difficult to follow. The manuscript does not consistently distinguish between independent and dependent variables in a manner that is easily understandable for general readers. While regression tables are presented, the narrative interpretation remains repetitive and fragmented. Greater clarity in explaining the analytical model and the relationships between variables would improve readability and comprehension.

• The discussion section tends to restate statistical findings rather than critically interpreting them within broader theoretical and public health contexts. Although the authors attempt to connect the findings to sociocultural dimensions of health literacy, the interpretation remains somewhat superficial. Stronger integration with existing literature, clearer theoretical implications, and deeper critical discussion are needed to enhance the scholarly contribution of the manuscript.

Reviewer #2: This manuscript is professional and clearly articulated. It follows the standard conventions of academic English quite well. The narrative structure is logical, successfully guiding the reader through the theoretical application of Bourdieu’s cultural capital within the specific context of Malaysian urban health literacy. While the prose is technically sound and free of major grammatical hurdles, it reads more like a standard research report than a high-impact discovery. It lacks the sharp, synthesis-driven storytelling that usually characterizes papers in top-tier journals. Instead, the text leans on descriptive phrasing that catalogs the findings rather than pushing a compelling or provocative new argument.

The clarity of the writing is certainly a strength because it makes the research easy to follow, but it does not manage to mask the study's incremental nature. The authors have done a solid job of presenting their data, but the writing does not offer the kind of big picture perspective or breakthrough narrative required to move the needle in global public health discourse. Ultimately, while the paper is linguistically precise and meets the basic requirements for an academic publication, it lacks the stylistic spark and transformative depth expected for a high-impact submission.

**********

6. PLOS authors have the option to publish the peer review history of their article (what does this mean?). If published, this will include your full peer review and any attached files.

If you choose “no”, your identity will remain anonymous but your review may still be made public.

**Do you want your identity to be public for this peer review?**  For information about this choice, including consent withdrawal, please see our Privacy Policy.

Reviewer #1: No

Reviewer #2: No

**********

[NOTE: If reviewer comments were submitted as an attachment file, they will be attached to this email and accessible via the submission site. Please log into your account, locate the manuscript record, and check for the action link "View Attachments". If this link does not appear, there are no attachment files.]

To ensure your figures meet our technical requirements, please review our figure guidelines: https://journals.plos.org/plosone/s/figures

You may also use PLOS’s free figure tool, NAAS, to help you prepare publication quality figures: https://journals.plos.org/plosone/s/figures#loc-tools-for-figure-preparation.

NAAS will assess whether your figures meet our technical requirements by comparing each figure against our figure specifications.

# Author comment: Author response to Decision Letter 1

**DOI:** 10.1371/journal.pone.0352662.r002

6 Jun 2026

Response to Reviewers

Manuscript title:

Cultural capital and health literacy among urban communities in Malaysia: A cross-sectional study

Dear Academic Editor and Reviewers,

We sincerely thank the reviewers for their insightful comments and constructive feedback. We have carefully revised the manuscript in response to these comments. Below we provide a detailed, point-by-point response. Changes have been incorporated throughout the manuscript as indicated.

General overview of revisions

We revised the introduction to provide a clearer explanation of Bourdieu’s forms of cultural capital and how each relates to health literacy. We sharpened the theoretical framework and research rationale, clarified the sampling strategy and sample size justification, described the study setting in greater detail and added a discussion of measurement development and limitations. The statistical analysis section was expanded to explain the choice of tests and assessment of regression assumptions. We also enhanced the discussion by linking findings to existing literature and policy implications. The references have been cross-checked and updated.

Responses to editorial assessment questions

1. Technical soundness and support for conclusions

Reviewer comment: Both reviewers indicated that the manuscript was partly technically sound and that the data partly supported the conclusions.

Response: We have revised the manuscript to ensure that the conclusions are more directly supported by the data. We now describe the study as cross-sectional and associative throughout the manuscript and avoid causal language. The conclusion has been revised to state that cultural capital was associated with health literacy domains, rather than implying causation. The limitations section now clearly acknowledges the cross-sectional design, convenience recruitment within clusters, limited generalisability, measurement limitations and the final sample size shortfall relative to the initial survey target.

2. Statistical analysis

Reviewer comment: Both reviewers indicated that the statistical analysis was not performed appropriately and rigorously.

Response: We have expanded the statistical analysis section to explain the analytical choices and assumption checks. Normality was assessed using the Shapiro-Wilk test and Spearman’s rank correlation was used for bivariate analyses because the study variables were not normally distributed. Multiple linear regression was retained for the multivariable analyses because the aim was to estimate the independent contribution of embodied, objectified and institutionalised cultural capital to each health literacy domain while reporting standardised coefficients and explained variance. We also added that regression assumptions were assessed, including linearity, residual distribution, homoscedasticity, multicollinearity and influential observations. Unstandardised coefficients, standardised beta coefficients, 95% confidence intervals, p-values and R² values are now reported.

3. Data availability

Reviewer comment: Both reviewers indicated that the data underlying the manuscript findings had not been made fully available.

Response: We have revised the Data Availability Statement. The anonymised dataset underlying the findings will be provided as Supporting Information. The dataset contains no personally identifiable information and includes the variables required to reproduce the descriptive, reliability, correlation and regression analyses reported in the manuscript.

4. Presentation and language

Reviewer comment: Reviewer 1 indicated that the manuscript was not sufficiently clear, while Reviewer 2 considered the manuscript clearly written but noted that it lacked a stronger high-impact narrative.

Response: We have edited the manuscript for clarity, flow and standard academic English. We clarified key concepts, reduced repetitive phrasing and improved transitions between the introduction, methods, results and discussion. We also strengthened the narrative by emphasising the broader public health significance of examining cultural capital as a sociocultural determinant of health literacy, particularly in contemporary information environments where access to information alone may not ensure meaningful evaluation or application.

Reviewer 1

Comment 1: Explanation of cultural capital

Reviewer comment: The manuscript introduces the concept of cultural capital based on Bourdieu’s framework; however, the explanation remains highly abstract and insufficiently contextualized for a multidisciplinary readership. Although embodied, objectified and institutionalized cultural capital are briefly defined, the manuscript does not adequately explain how these constructs practically relate to health literacy in everyday settings. Readers outside sociology or health promotion may find it difficult to comprehend the meaning and application of these concepts within the study context. More concrete examples and clearer operational explanations are needed to improve accessibility and interpretability of the findings.

Response: We agree with this comment. The introduction has been revised to explain the three forms of cultural capital in more concrete terms. Institutionalised cultural capital is now explained as formal educational qualifications; objectified cultural capital as material and informational resources, including digital devices and health information sources; and embodied cultural capital as durable dispositions, confidence, communication practices, experiential knowledge and practical judgement. We also added examples of how these forms may relate to health literacy, such as formal education supporting comprehension of written health information, material resources increasing exposure to health information and embodied dispositions shaping confidence and judgement in health-related decision-making.

Comment 2: Theoretical framework

Reviewer comment: The study lacks a robust theoretical framework underpinning the proposed relationship between cultural capital and health literacy. While Bourdieu’s theory is cited, the manuscript does not sufficiently explain the theoretical mechanisms linking each form of cultural capital to the domains of health literacy. The rationale for why institutionalized cultural capital should influence access and understanding, or why embodied cultural capital predicts evaluation and application, is underdeveloped. As a result, the study appears largely descriptive and correlational rather than theory-driven.

Response: We have strengthened the theoretical framing in the introduction and discussion. The revised manuscript now explains why institutionalised cultural capital may be more closely linked to access and understanding, while embodied cultural capital may be more important for evaluation and application. We explain that access and understanding may depend more on formal education and familiarity with written or institutional information, whereas evaluation and application require practical judgement, confidence, prior experience and the ability to decide whether information is credible and relevant to one’s circumstances. This makes the manuscript more theory-driven and less purely descriptive.

Comment 3: Study rationale and Malaysian context

Reviewer comment: The rationale for conducting the study requires further strengthening. Although the introduction discusses increasing access to health information and persistent disparities in health literacy, the manuscript does not clearly establish the specific research gap that justifies this study within the Malaysian context. The reasons for focusing specifically on urban communities in Perak are not adequately explained. The manuscript would benefit from a clearer justification regarding the public health importance, contextual relevance, and potential implications of examining cultural capital within urban Malaysian populations.

Response: We have revised the introduction to clarify the research gap. The manuscript now explains that although access to health information has expanded in Malaysia, disparities in health literacy persist. Existing studies have largely focused on education, digital access or individual literacy skills, with less attention to the sociocultural resources that may shape higher-order health literacy competencies such as evaluation and application. We also strengthened the justification for the study setting. Ipoh, Perak is now described as an urban Malaysian setting with a socially diverse population and variation in education, income, employment and material resources. This provides a relevant context for examining how cultural capital relates to health literacy.

Comment 4: Sampling rigour and representativeness

Reviewer comment: The methodological approach raises concerns regarding sampling rigor and representativeness. The manuscript describes the use of a “multistage cluster sampling approach”; however, participant recruitment within clusters was ultimately conducted using convenience sampling. This creates ambiguity regarding the actual sampling strategy and may overstate the methodological robustness of the study. The use of convenience sampling limits representativeness and introduces potential selection bias, which should be more explicitly acknowledged and discussed.

Response: We agree that this required clarification. The methods section has been revised to state that urban localities were defined as clusters based on geographical and administrative boundaries, but that eligible participants within these clusters were recruited through community-based convenience sampling because no comprehensive household-level or individual-level sampling frame was available. We also added a clear statement that the sample should not be interpreted as statistically representative of all urban residents in Perak. The limitations section now acknowledges that this recruitment approach may have introduced selection bias because participation depended on availability and willingness to participate at the time of data collection.

Comment 5: Sample size justification

Reviewer comment: The manuscript does not provide any evidence or rationale explaining how the sample size of 325 respondents was determined. No power analysis, sample size formula, effect size estimation, or reference to regression sample size requirements is presented. Given that multiple regression analyses were conducted, an appropriate justification for the adequacy of the sample size is necessary to support the validity of the statistical findings.

Response: We have added a sample size justification in the methods section. The initial target sample size was guided by Cochran’s single-proportion formula for cross-sectional studies, using a 95% confidence level, an expected proportion of 50% and a 5% margin of error, which gave a minimum target of 384 respondents. The final sample included 325 respondents after data screening. We acknowledge that this was below the initial target. However, the sample remained adequate for the planned multiple regression analyses, as each model included three predictors and Green’s recommendation of N > 50 + 8m requires a minimum of 74 respondents for three predictors. We have also added this issue to the limitations section by stating that the final sample of 325 respondents was smaller than the minimum target of 384 calculated with Cochran’s formula; while adequate for the planned regression analyses, the shortfall may reduce the precision of estimates and limit statistical power.

Comment 6: Study setting

Reviewer comment: The description of the study setting lacks sufficient detail. The term “selected urban communities” is repeatedly used without adequately describing the characteristics of these communities. It remains unclear whether the sample included urban poor populations, middle-income groups, highly educated residents, or mixed socioeconomic backgrounds. Since cultural capital is closely linked to social and economic positioning, clearer contextual description of the study population is essential for interpretation and transferability of the findings.

Response: We have expanded the “Study design and setting” section. The selected communities are now described as urban residential areas with mixed socioeconomic characteristics, including variation in educational attainment, employment status, income and exposure to health information sources. We also explain that this diversity is important because cultural capital is closely linked to social position and may vary across educational and socioeconomic groups. The demographic table and narrative results further describe the sample in terms of age, gender, ethnicity, education, employment and income.

Comment 7: Operationalisation and validation of cultural capital

Reviewer comment: The operationalization of cultural capital requires further clarification and validation. Although the authors state that measurement items were developed based on the conceptual framework and adapted to the study context, there is insufficient information regarding item development procedures, construct validity assessment, dimensionality testing, or validation processes beyond Cronbach’s alpha values. Reliability alone is insufficient to establish the validity of a newly operationalized construct, particularly for a complex sociological concept such as cultural capital.

Response: We have expanded the measures section to describe how cultural capital was operationalised. The revised manuscript explains that items were developed by mapping Bourdieu’s conceptual categories to observable indicators relevant to health information engagement. Embodied cultural capital included indicators related to confidence in discussing health information, experience interpreting health advice and familiarity with using health information in everyday decision-making. Objectified cultural capital represented access to health-related materials and digital or informational resources, while institutionalised cultural capital represented formal educational qualifications. The items were reviewed by the research team for conceptual relevance and contextual suitability and were pre-tested for clarity, wording and acceptability. We also clarified that Cronbach’s alpha demonstrates internal consistency but does not establish full construct validity. The limitations section now states that future studies should examine dimensionality and construct validity using exploratory and confirmatory factor analysis and validate the instrument across different Malaysian populations.

Comment 8: Presentation of results

Reviewer comment: The presentation of the results is statistically organized but conceptually difficult to follow. The manuscript does not consistently distinguish between independent and dependent variables in a manner that is easily understandable for general readers. While regression tables are presented, the narrative interpretation remains repetitive and fragmented. Greater clarity in explaining the analytical model and the relationships between variables would improve readability and comprehension.

Response: We have revised the results section to improve conceptual clarity. The manuscript now explicitly states that embodied, objectified and institutionalised cultural capital were entered as independent variables, while access, understanding, evaluation and application were analysed as dependent variables in separate regression models. We also explain that unstandardised coefficients represent the expected change in the outcome for a one-unit increase in the predictor, while standardised coefficients allow comparison of relative predictor strength within each model. The narrative now focuses on the differentiated pattern across health literacy domains rather than merely listing statistical values.

Comment 9: Discussion and interpretation

Reviewer comment: The discussion section tends to restate statistical findings rather than critically interpreting them within broader theoretical and public health contexts. Although the authors attempt to connect the findings to sociocultural dimensions of health literacy, the interpretation remains somewhat superficial. Stronger integration with existing literature, clearer t

Attachment Submitted filename: Response to Reviewers.docx

# Peer review: Decision Letter 1

**DOI:** 10.1371/journal.pone.0352662.r003

**Authors:** Kristiawan Indriyanto, Kristiawan Indriyanto

14 Jun 2026

Cultural capital and health literacy among urban communities in Malaysia: A cross-sectional study

PONE-D-26-16700R1

Dear Dr. Ab Rahman,

We’re pleased to inform you that your manuscript has been judged scientifically suitable for publication and will be formally accepted for publication once it meets all outstanding technical requirements.

Within one week, you’ll receive an e-mail detailing the required amendments. When these have been addressed, you’ll receive a formal acceptance letter and your manuscript will be scheduled for publication.

An invoice will be generated when your article is formally accepted. Please note, if your institution has a publishing partnership with PLOS and your article meets the relevant criteria, all or part of your publication costs will be covered. Please make sure your user information is up-to-date by logging into Editorial Manager at Editorial Manager® and clicking the ‘Update My Information' link at the top of the page. For questions related to billing, please contact billing support.

If your institution or institutions have a press office, please notify them about your upcoming paper to help maximize its impact. If they’ll be preparing press materials, please inform our press team as soon as possible -- no later than 48 hours after receiving the formal acceptance. Your manuscript will remain under strict press embargo until 2 pm Eastern Time on the date of publication. For more information, please contact onepress@plos.org.

Kind regards,

Kristiawan Indriyanto

Academic Editor

PLOS One

Additional Editor Comments (optional):

Reviewers' comments:

Reviewer's Responses to Questions

**Comments to the Author**

1. If the authors have adequately addressed your comments raised in a previous round of review and you feel that this manuscript is now acceptable for publication, you may indicate that here to bypass the “Comments to the Author” section, enter your conflict of interest statement in the “Confidential to Editor” section, and submit your "Accept" recommendation.

Reviewer #1: All comments have been addressed

**********

2. Is the manuscript technically sound, and do the data support the conclusions?

The manuscript must describe a technically sound piece of scientific research with data that supports the conclusions. Experiments must have been conducted rigorously, with appropriate controls, replication, and sample sizes. The conclusions must be drawn appropriately based on the data presented.

Reviewer #1: Yes

**********

3. Has the statistical analysis been performed appropriately and rigorously?

Reviewer #1: Yes

**********

4. Have the authors made all data underlying the findings in their manuscript fully available?

The PLOS Data policy requires authors to make all data underlying the findings described in their manuscript fully available without restriction, with rare exception (please refer to the Data Availability Statement in the manuscript PDF file). The data should be provided as part of the manuscript or its supporting information, or deposited to a public repository. For example, in addition to summary statistics, the data points behind means, medians and variance measures should be available. If there are restrictions on publicly sharing data—e.g. participant privacy or use of data from a third party—those must be specified.

Reviewer #1: Yes

**********

5. Is the manuscript presented in an intelligible fashion and written in standard English?

PLOS ONE does not copyedit accepted manuscripts, so the language in submitted articles must be clear, correct, and unambiguous. Any typographical or grammatical errors should be corrected at revision, so please note any specific errors here.

Reviewer #1: Yes

**********

6. Review Comments to the Author

Please use the space provided to explain your answers to the questions above. You may also include additional comments for the author, including concerns about dual publication, research ethics, or publication ethics. (Please upload your review as an attachment if it exceeds 20,000 characters)

Reviewer #1: The revised manuscript demonstrates substantial improvement in theoretical framing, methodological transparency, statistical reporting, and discussion. The authors have adequately addressed the major concerns raised during the previous review round. I therefore consider the manuscript suitable for publication in its current form, subject to minor editorial revisions.

**********

7. PLOS authors have the option to publish the peer review history of their article (what does this mean?). If published, this will include your full peer review and any attached files.

If you choose “no”, your identity will remain anonymous but your review may still be made public.

**Do you want your identity to be public for this peer review?**  For information about this choice, including consent withdrawal, please see our Privacy Policy.

Reviewer #1: **Yes:** Dr Mohd Ismail Ibrahim

**********

# Editor report: Acceptance letter

**DOI:** 10.1371/journal.pone.0352662.r004

**Authors:** Kristiawan Indriyanto, Kristiawan Indriyanto

PONE-D-26-16700R1

PLOS One

Dear Dr. Ab Rahman,

I'm pleased to inform you that your manuscript has been deemed suitable for publication in PLOS One. Congratulations! Your manuscript is now being handed over to our production team.

At this stage, our production department will prepare your paper for publication. This includes ensuring the following:

* All references, tables, and figures are properly cited

* All relevant supporting information is included in the manuscript submission,

* There are no issues that prevent the paper from being properly typeset

You will receive further instructions from the production team, including instructions on how to review your proof when it is ready. Please keep in mind that we are working through a large volume of accepted articles, so please give us a few days to review your paper and let you know the next and final steps.

Lastly, if your institution or institutions have a press office, please let them know about your upcoming paper now to help maximize its impact. If they'll be preparing press materials, please inform our press team within the next 48 hours. Your manuscript will remain under strict press embargo until 2 pm Eastern Time on the date of publication. For more information, please contact onepress@plos.org.

You will receive an invoice from PLOS for your publication fee after your manuscript has reached the completed accept phase. If you receive an email requesting payment before acceptance or for any other service, this may be a phishing scheme. Learn how to identify phishing emails and protect your accounts at https://explore.plos.org/phishing.

If we can help with anything else, please email us at customercare@plos.org.

Thank you for submitting your work to PLOS ONE and supporting open access.

Kind regards,

PLOS ONE Editorial Office Staff

on behalf of

Dr. Kristiawan Indriyanto

Academic Editor

PLOS One
